# Exclusive color-coded duplex sonography of extracranial vessels reliably confirms brain death: A prospective study

**DOI:** 10.3389/fneur.2022.992511

**Published:** 2022-09-23

**Authors:** Johann Lambeck, Christoph Strecker, Wolf-Dirk Niesen, Jürgen Bardutzky

**Affiliations:** Department of Neurology and Clinical Neurophysiology, Freiburg University Medical Center, Freiburg, Germany

**Keywords:** cerebral circulatory arrest ultrasound, electrocerebral inactivity, ancillary test, irreversible loss of brain function, neck vessels brain death

## Abstract

**Background:**

Transcranial color-coded duplex sonography (TCCD) can be used as an ancillary test for determining irreversible loss of brain function (ILBF) when demonstration of cerebral circulatory arrest (CCA) is required. However, visualization of the intracranial vessels by TCCD is often difficult, or even impossible, in this patient cohort due to elevated intracranial pressure, an insufficient transtemporal bone window, or warped anatomical conditions. Since extracranial color-coded duplex sonography (ECCD) can be performed without restriction in the aforementioned situations, we investigated the feasibility of omitting TCCD altogether, such that the ILBF examination would be simplified, without compromising on its reliability.

**Methods:**

A total of 122 patients were prospectively examined by two experienced neurointensivists for the presence of ILBF from 01/2019-12/2021. Inclusion criteria were (i) the presence of a severe cerebral lesion on cranial CT or MRI, and (ii) brainstem areflexia. Upon standardized clinical examination, 9 patients were excluded due to incomplete brainstem areflexia, and a further 22 due to the presence of factors with a potentially confounding influence on apnea testing, EEG or sonography. A total of 91 patients were enrolled and underwent needle-EEG recording for >30 min (= gold standard), as well as ECCD and TCCD. The sonographer was blinded to the EEG result.

**Results:**

All patients whose ECCD result was consistent with ILBF had this diagnosis confirmed by EEG (*n* = 77; specificity: 1). Both ECCD and EEG were not consistent with ILBF in a further 12 patients. In the remaining two patients, ECCD detected reperfusion due to long-lasting cerebral hypoxia; however, ILBF was ultimately confirmed by EEG (sensitivity: 0.975). This yielded a positive predictive value (PPV) of one and a negative predictive value of 0.857 for the validity of ECCD in ILBF confirmation. TCCD was not possible/inconclusive in 31 patients (34%).

**Conclusions:**

The use of ECCD for the confirmation of ILBF is associated with high levels of specificity and a high positive predictive value when compared to needle-electrode EEG. This makes ECCD a potential alternative to the ancillary tests currently used in this setting, but confirmation in a multi-center trial is warranted.

**Trial registration:**

https://www.drks.de, DRKS00017803.

## Introduction

According to various international guidelines, the determination of irreversible loss of brain function (i.e., brain death, ILBF) is a multistage process (1–3). Despite efforts by the World Health Organization (WHO) to harmonize international recommendations for determining ILBF, the exact procedure and application of ancillary tests still vary from country to country, and in some cases, even within countries (4, 5). The German Medical Association (GMA) guideline (6) recommends the following conditions for establishing ILBF: (i) the presence of a severe brain injury that serves as a sufficient basis for cerebral circulatory arrest (CCA) due to increased intracranial pressure (ICP), in combination with a comatose state of consciousness. For the latter, alternative and possibly reversible causes [i.e., intoxication, analgosedation (7, 8), hypothermia, metabolic coma, circulatory shock] must have already been ruled out; (ii) ascertainment of brainstem areflexia, including the loss of spontaneous breathing. In this instance, (iii) the irreversibility of the complete loss of whole-brain function must be demonstrated. Indeed, it is possible to determine ILBF by the use of various ancillary tests (9), or by the repetition of the clinical examination after a certain observation period; however, this depends on the patient group, country, ILBF concept and guidelines. Some guidelines deem the use of ancillary testing mandatory as a matter of principle (10), or at least under certain circumstances; this particularly holds true in the case of primary infratentorial cerebral lesions, since the clinical diagnosis focuses on brain stem areflexia (6). The latter may well-be explained by the focal process occurring in this patient group, where partially or completely preserved supratentorial cerebral function is possible (concepts of whole brain vs. brain stem death).

With regard to ancillary testing, there are a number of different, internationally accepted tests that focus on demonstrating either the loss of brain function (i.e., EEG and evoked potentials) or CCA (i.e., transcranial doppler or color-coded duplex sonography of the cerebral vessels [TCD and TCCD], catheter angiography, CT-angiography, SPECT) (11–13). Several international neurophysiological societies have published concise recommendations on the technical prerequisites for the application of these techniques in the context of ILBF (14–16). In terms of TCCD, the visualization of the intracranial vessels using this technique is often difficult or impossible due to an elevated ICP, the absence of a transtemporal bone window in nearly 10 % of patients (17, 18), the warped anatomical conditions that arise from cerebral injury, hemorrhages, as well as edematous swelling.

Previous studies in this context have suggested that extracranial color-coded duplex sonography (ECCD) can be applied alone or in combination with TC (C)D (19–22), whereby ECCD has a sensitivity level of 78% (22). The combination of ECCD with TCCD has been shown to increase sensitivity up to 100% (20). However, to our best knowledge, the prospective dataset is thus far only limited to 20 patients (20). The advantages of ECCD are that (i) it relies neither on the presence of an adequate bone window, particularly in older patients (21), nor a specially trained operator (22), and (ii) it allows the direct visualization of the vessel lumen (20).

The present study therefore prospectively investigated whether omitting TCCD and solely carrying out bilateral color-coded duplex sonography of the extracranial carotid and vertebral arteries would be feasible to simplify the ILBF examination without worsening its reliability, which is of paramount importance. ECCD results were compared to those of a needle EEG which was used as the gold standard technique to determine the irreversibility of brain function loss (primary endpoint: demonstration of non-inferiority of ECCD alone vs. EEG [gold standard] in the confirmation of ILBF).

## Materials and methods

### Ethics check

This study was approved by our local ethics committee (Nr. 368/19) and registered at the German Clinical Trials Register (DRKS00017803). It was performed in accordance with the ethical standards laid down in the 1964 Declaration of Helsinki and its later amendments. The full study protocol can be accessed via the DRKS website (www.drks.de). In all cases, informed consent was obtained from the patient's relatives.

### Study population

A group of 122 consecutive patients with severe brain damage of various etiology (Table 1) was prospectively examined between January 2019 and December 2021 for the presence of ILBF. Study inclusion criteria were: the presence of a severe cerebral lesion (as evidenced by cranial CT or MRI) and brainstem areflexia. Exclusion criteria comprised factors that potentially influence EEG (i.e., relevant levels of analgosedatives), apnea test (i.e., relevant levels of analgosedatives and/or relevant COPD) or duplex sonography (i.e., large osseus defects, or low cardiac output in vaECMO-patients). Furthermore, patients with incomplete brainstem areflexia were excluded.

**Table 1:** Patient characteristics of all 122 screened patients and further details of the ILBF examinations.

Age (years)	median 54, IQR 42–64,75
Sex (female/male)	50 / 72
Screened patients	122
**Etiology of brain lesion**	
ICH	21
SAH	11
Ischemic Stroke	12
TBI	3
Hypoxia	40
Combined	28
Other	7
Included patients	91
Excluded patients	31
Incomplete brainstem areflexia	9
Large cranial osseus defects	8
Cardiac output too low (vaECMO)	5
Relevant levels of analgosedatives	6
Relevant COPD	3
ILBF confirmed	79
ILBF not confirmed	12

ILBF, irreversible loss of brain function; IQR, interquartile range; ICH, intracerebral hemorrhage; SAH, subarachnoid hemorrhage; TBI, traumatic brain injury; vaECMO, veno-arterial extracorporeal membrane oxygenation; COPD, chronic obstructive pulmonary disease.

A target sample size of at least 100 patients was calculated based on the number of ILBF candidates previously examined per annum.

### Clinical assessment

In our University Medical Center, brain death is diagnosed in accordance with the guidelines published by the German Medical Association (GMA) by a team of highly specialized and experienced neurointensivsts (i.e., each board-certified in neurology and intensive care medicine, several years of experience in both the clinical examination of brain dead patients and ancillary testing and more than 100 cases each). This team is consulted in all cases of ICU-patients within the University Medical Center Freiburg with clinically suspected brain death (i.e., severe brain damage on cranial CT or MRI, pupils fixed and dilated, apnea), mainly in the context of organ donation, but also in the context of end-of-life care and decision-making concerning the continuation or cessation of intensive care measures. All examinations were performed >24 h after initial detection of the above-mentioned clinical signs of brain death.

For each patient, a thorough review of the case, including the examination of all available cerebral imaging and laboratory data was initially performed. After ruling out alternative factors that could explain either in whole or in part the patient's comatose state of consciousness (e.g., sedation, shock, etc.), clinical assessment of brainstem reflexes, including apnea testing, was performed.

Patients with incomplete brain stem areflexia or with clinically relevant levels of analgosedatives (and therefore potentially altered EEG findings) as well as those with clinically relevant COPD [i.e., adaptation to elevated levels of CO_2_, as demonstrated by blood gas analysis: (i) paCO_2_ outside the required 35-45 mmHg range, (ii) simultaneous pH range of 7.35–7.45, (iii) altered base excess] were subsequently excluded from the study. In the latter subgroup, GMA guidelines require demonstration of CCA and EEG is not allowed.

### ILBF assessment (EEG)

In all remaining patients, and in accordance with GMA guidelines (6, 14), an EEG (Deltamed itmed^®^ machine with Neurofile^®^ software on a Lenovo ThinkPad^®^ laptop computer, 23 steel-needle electrodes, 10–20 placement, electrode impedance 1–5 kΩ, high pass filter 70 Hz, low pass filter 0.53–0.16 Hz/time constant 0.3–1 s, amplification 2 μV/mm, repeated application of painful stimuli to the face and extremities, auditory and visual stimuli, additional double distance montage) was recorded after clinical assessment by one of the two examiners for > 30 min to confirm irreversibility of the condition. Short-acting muscle relaxants (e.g. rocuronium i.v.) were applied in patients with residual scalp EMG activity. Recording of a flatline EEG (i.e., electrocerebral inactivity) over more than 30 min is required to confirm ILBF. EEG was chosen as the reference standard for the following reasons: (i) EEG is a bedside test, (ii) EEG does not require transport of instable patients to diagnostic facilities, (iii) EEG is applicable in most ILBF candidates since there are no restrictions related to age or lesion pattern/mechanism according to GMA regulations.

### ILBF assessment (color-coded duplex sonography)

**Figure 1:**
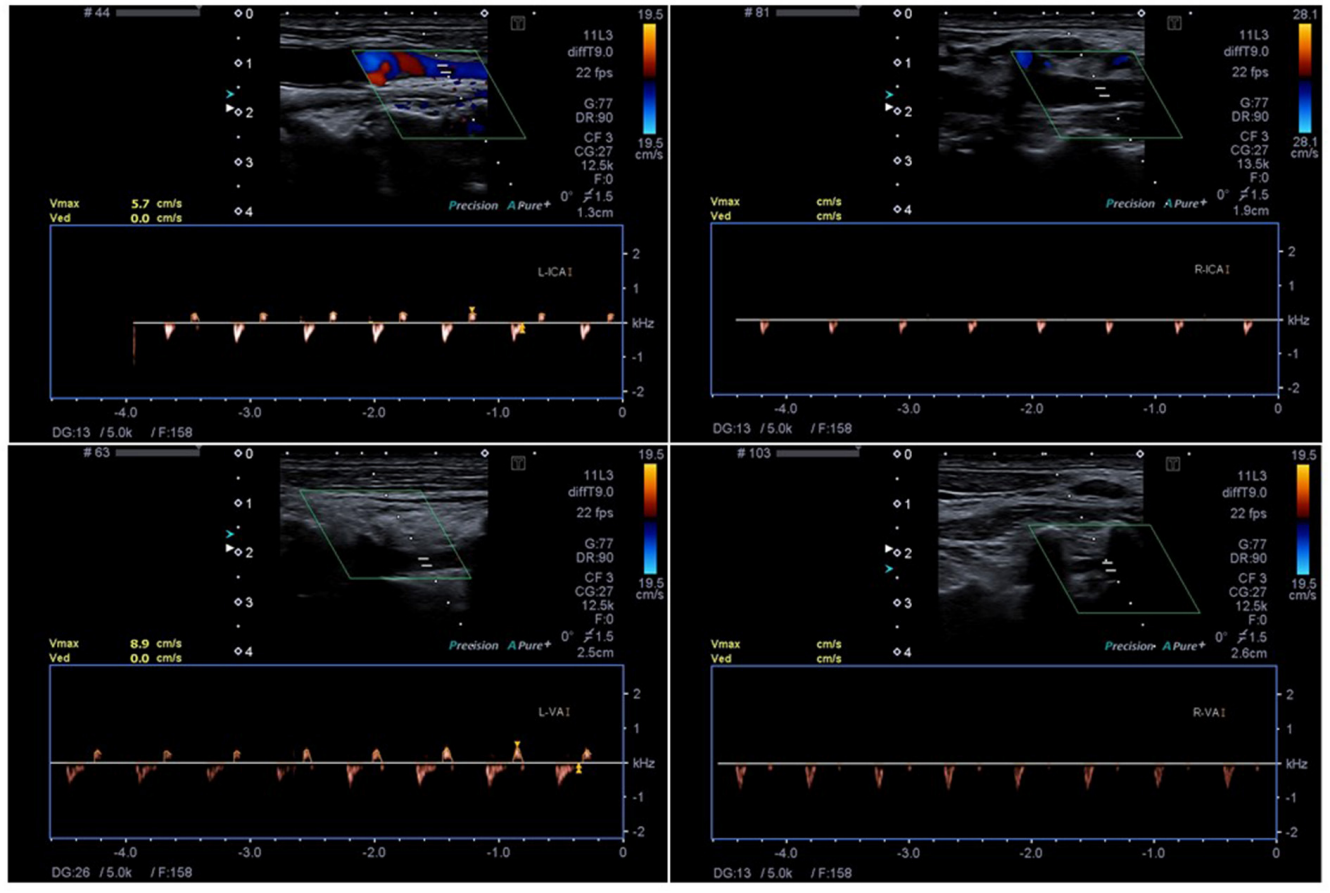
The Technique of ECCD with detection of small systolic spikes in the right ICA (R-ICA) and VA (R-VA) and biphasic flow in the left ICA (L-ICA) and VA (L-VA). The insonation depth is 1.9 cm (R-ICA), 2.6 cm (R-VA), 1.3 cm (L-ICA) and 2.5 cm (L-VA), respectively.

The second examiner (who was blinded to the result of the EEG) directly subsequently performed a color-coded duplex sonographic examination (Philips CX50^®^ or Toshiba Aplio^®^ 400, L12-3 broadband linear array transducer, frequency spectrum 3–12 MHz and S5-1 Broadband pure wave sector array transducer, frequency spectrum 1–5 MHz, Figure 1). The GMA and DGKN-guidelines ask for the demonstration of CCA signs, i.e., early systolic peaks or biphasic “pendulum” flow with equal antero- and retrograde parts of the doppler time frequency spectrum within one cardiac cycle) for more than 30 min to confirm ILBF (i.e., recording of the below mentioned vessel sections at the beginning and just after a 30 min time-span) (6, 14). In patients with biphasic flow signals, care was taken to only accept narrow, monophasic flow signals (orthograde component). A mean arterial pressure of > 60 mmHg is required by GMA guidelines, in patients who did not meet this, noradrenaline was administered i.e., the same prerequisites were applied to the ECCD examination. This also extended to guideline conformity: in patients with large osseus defects, regionally limited cerebral circulation may exist (e.g., via extra-/intracranial anastomoses). In these patients, CCA cannot be demonstrated by TCCD of the basal cerebral arteries. Since the same criteria were applied to our study cohort, these patients were excluded from the study. Furthermore, in some of the patients undergoing vaECMO treatment, sonographic ascertainment of pulsatile vascular flow signals was not possible due to low cardiac output. Therefore, these patients were also excluded from the study.

TCCD included bilateral examination of the middle cerebral artery (M1 segments), internal carotid artery (ICA, C1 segment), vertebral artery (VA, V4/5 segment) and examination of the basilar artery (BA) and all other visible intracranial arteries. ECCD included bilateral examination of the ICA) as distally as possible and the V2/3 segment of the VA, respectively.

### Primary endpoint

The primary endpoint was to show that the use of ECCD alone to confirm ILBF is not inferior to the use of gold-standard EEG in the same context.

#### Statistical analysis and data presentation

Statistical analyses (specificity, sensitivity, positive and negative predictive values) were performed using the IBM^®^ SPSS^®^ Statistics 21 software package (IBM Corporation, Armonk, NY). Data were found to be non-normally distributed and are presented as median and interquartile range (IQR).

## Results

### Patient characteristics

**Figure 2:**
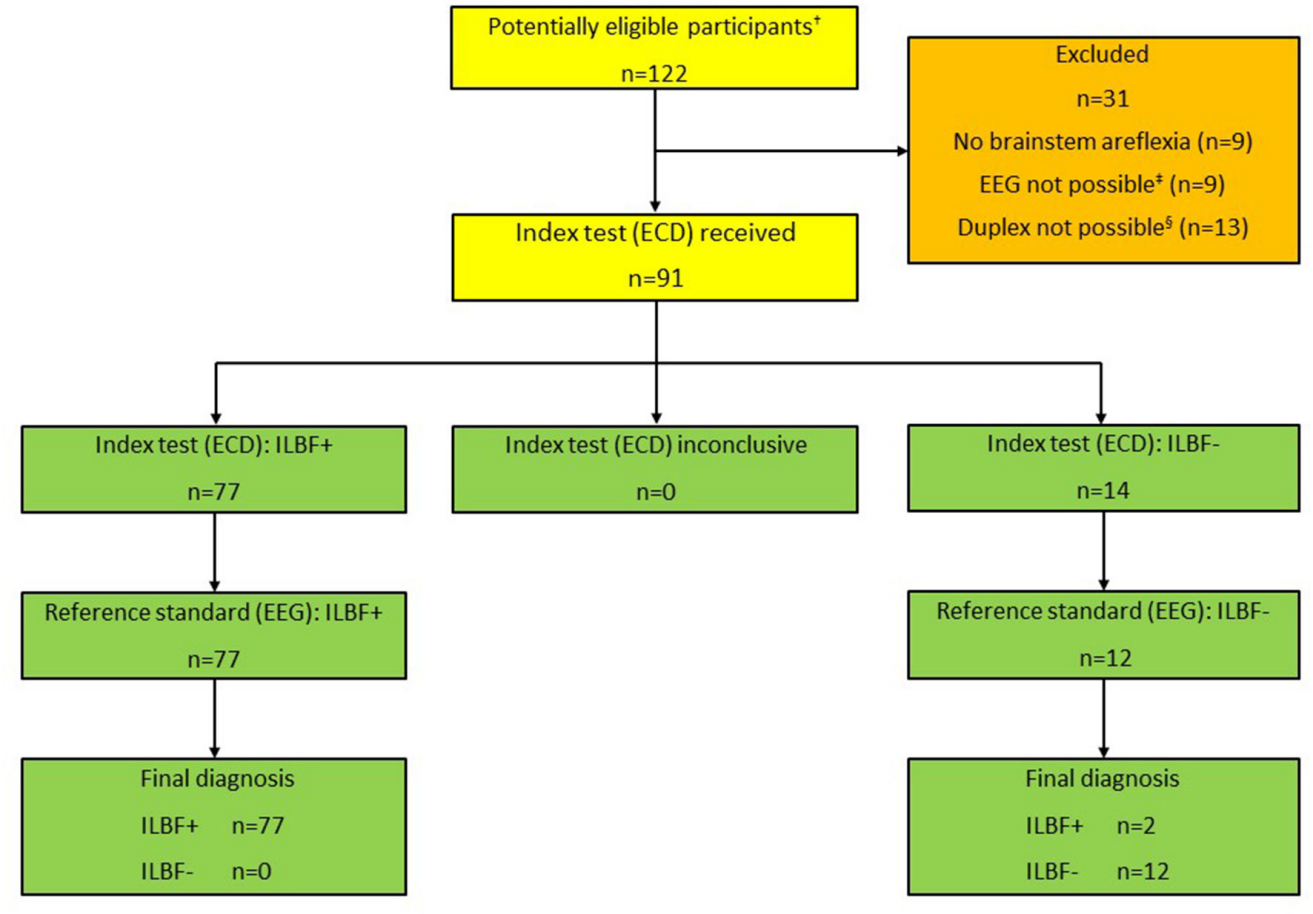
Patient inclusion scheme, all patients (*n* = 122). ^†^Total number of patients that were examined as ILBF candidates 01/2019-12/2021. ^‡^Due to relevant levels of analgosedatives (*n* = 6), relevant COPD (*n* = 3). ^§^Due to large cranial osseus defects (*n* = 8), low cardiac output (vaECMO; *n* = 5). ILBF+ Test result consistent with irreversible loss of brain function. ILBF- Test result not consistent with irreversible loss of brain function.

A total of 122 ILBF examinations were performed from 01/2019 to 12/2021. The patient characteristics of all patients and further details of the ILBF examinations are shown in Table 1. The inclusion scheme (following the STARD recommendations) is shown in Figure 2.

Incomplete brain stem areflexia was detected in nine patients. EEG was not possible in a total of nine patients, due to clinically relevant levels of analgosedatives in six and COPD in three patients. In these cases, the GMA guideline requires demonstration of CCA due to a potential influence on EEG and/or apnea testing.

Sonography was not allowed (guideline inconformity) or impossible in a total number of 13 patients. This was due to large osseus defects (*n* = 8 patients) or low cardiac output in patients on veno-arterial ECMO/ECLS (*n* = 5).

### ILBF testing

#### Feasibility

In the cohort of 111 patients with brain stem areflexia, EEG was allowed and feasible in 104 patients (94%); none of the EEG recordings was rendered unusable due to artifact. Duplex sonography was allowed in 98 patients, in all of whom ECCD was also feasible (88% of all patients). In 91 patients, both EEG and duplex sonography were allowed for ILBF confirmation. No adverse events occurred due to the application of the reference standard or the study exam.

#### ECCD

In all patients with an ECCD result consistent with ILBF, EEG was also consistent with ILBF. ECCD was not consistent with ILBF in 14 patients, and EEG was also not consistent with ILBF in 12 out of these patients. In both remaining patients, ECCD detected cerebral (re-) perfusion. In both cases (one on vaECMO-therapy), this was due to long-lasting cerebral hypoxia. In both cases, however, EEG confirmed ILBF.

#### Test validity (ECCD)

**Figure 3:**
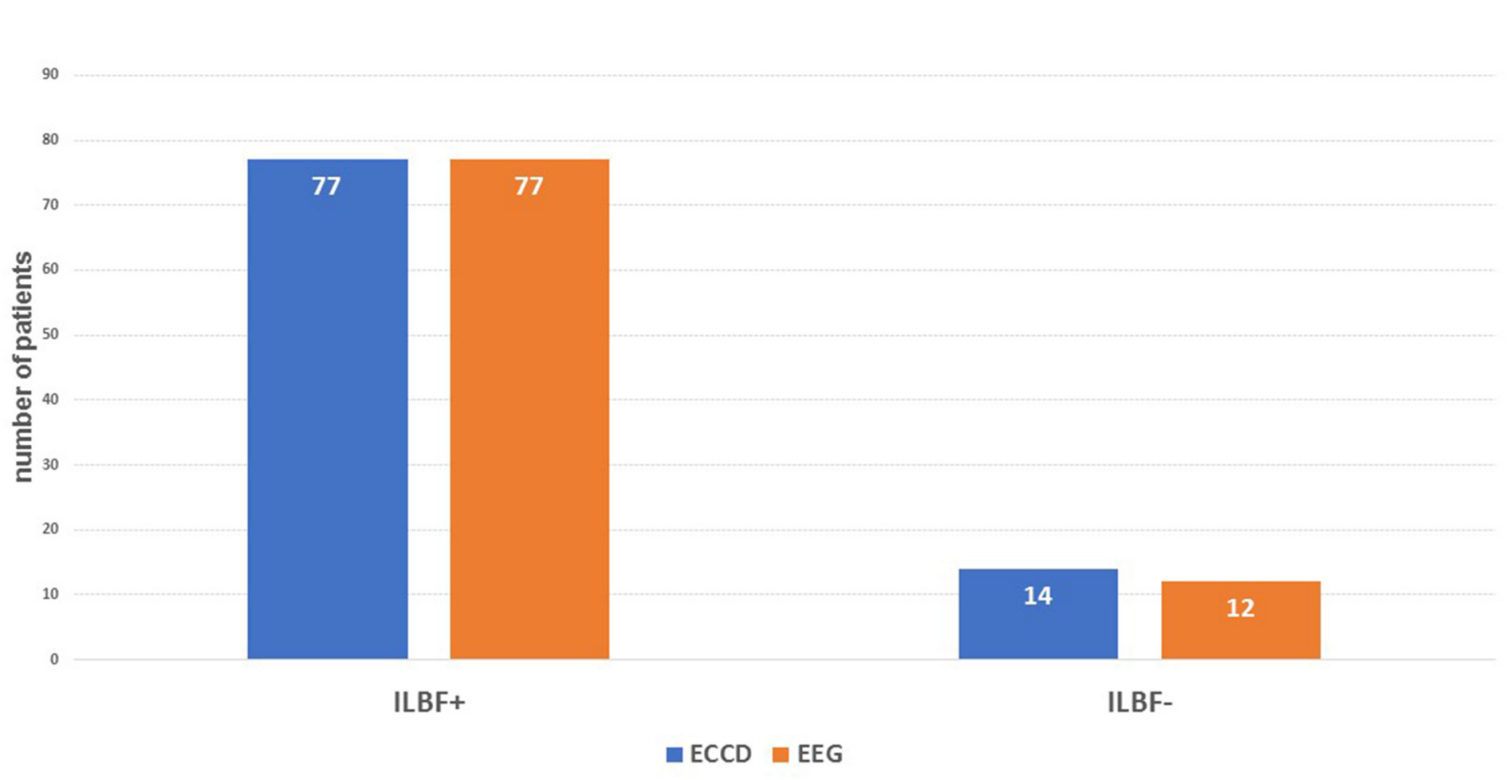
The main results of the index test (ECD) compared to the gold standard (EEG). ILBF+, result consistent with ILBF; ILBF-, result inconsistent with ILBF. The resulting specificity is 1.0, positive predictive value is 1.0, sensitivity is 0.975, and negative predictive value 0.857.

Concerning the validity of ECCD in ILBF confirmation in comparison to gold-standard EEG, this yielded a specificity level and positive predictive value (PPV) of one, a sensitivity level of 0.975, and a negative predictive value (NPV) of 0.857. There were hence no false positive and only two false negative results (see also Figure 3).

#### TCCD

TCCD was not possible/inconclusive in a total of 31 patients (34% of the examinations). This was specifically due to the absence of a sufficient temporal bone window in 10 patients, a gunshot wound in 1 patient, and the inability to detect intracranial vessels in 20 patients. In the 60 remaining patients, TCCD yielded the same results as ECCD; in particular, there were no cases in which TCCD showed CCA, but ECCD showed residual perfusion.

## Discussion

This study investigated the feasibility of using ECCD – without parallel application of TCCD – for confirming ILBF in patients with severe cerebral lesions. By comparing the results from ECCD alone with gold-standard EEG findings, it was found that exclusive duplex sonography of the extracranial cervical arteries that detects the typical signs of CCA is sufficient to demonstrate irreversible loss of brain function. In all cases where ECCD revealed findings consistent with ILBF, there was no detection of residual cerebral activity by EEG (specificity and positive predictive value = 1, Figure 3).

The high degree of ECCD validity in our study is similar to that reported in previous studies on the application of TC (C)D (18, 23, 24) in this context. The observed sensitivity was higher than that found in a retrospective study on ECCD (22). Visualization of all four neck vessels (bilateral ICA and VA) was possible in all 91 patients, which has also been shown in a previous prospective study on ECCD in 20 brain-death candidates; here, the supplementation of TCCD with ECCD increased the sensitivity to 100% (20). In our study, however, TCCD was not helpful in determining ILBF in 34% of cases, but there were no cases in which TCCD showed CCA, but ECCD did not.

In the present study, following complete clinical examination, EEG was used as the gold standard for determining ILBF. EEG was chosen as the reference test since the alternative ancillary tests either are not bedside and require transport to diagnostic facilities (CTA, DSA, SPECT) or are not permitted in certain age groups (CTA, SSEP) or in patients with infratentorial cerebral lesions (AEP, SSEP) according to GMA guidelines. Furthermore, to rule out any potential confounders that could influence both ECCD and TCCD, we chose an ancillary test as a comparator for demonstrating the loss of cerebral function rather than comparing two tests that both aim to demonstrate the loss of cerebral perfusion. In the context of ILBF determination, EEG validity has previously been reported as 0.94 (13), specificity as 0.97, and sensitivity as 0.804 (4) which is similar to our data. In line with the concept of “whole brain death,” the use of EEG (which mainly assesses cortical neuronal activity) is an ideal complement to clinical ILBF testing (with the focus on brainstem areflexia).

However, some studies have reported the detection of EEG activity up to several days after clinical diagnosis of brain death (25, 26) and susceptibility to ICU-related electromagnetic noise and other artifacts have been criticized (4, 27). Interobserver variability is also a disputed factor and has thus led to debates about its application as an ancillary test (4). EEG mainly assesses cortical function, so its application as an ancillary test is not useful if brainstem death is accepted as a concept. Brainstem function is assessed clinically, but patients fulfilling the clinical criteria for brain death may well-have partially preserved cortical activity (28). This is corroborated by histopathological studies that have shown relative preservation of the cerebral cortex in patients with clinical brain death syndrome and residual EEG activity (25, 28). Regarding artifact liability, the exclusive use of steel needle electrodes in our study resulted in a dramatic reduction in surface resistance at the skin-electrode interface (and hence artifact susceptibility). Accordingly, none of the EEGs performed in our study patients was precluded by artifacts. To reduce the problem of interobserver variability, we believe that recording an EEG in this context should be done by or at least in the presence of the intensivists themselves, and not by technical assistants with subsequent “offline” analysis by the physician. The former ensures that potential artifacts can be detected, attributed to a source and eliminated directly.

In all but two cases where the ECCD result was inconsistent with ILBF (*n* = 14), this was corroborated by EEG findings. Both the patients with preserved cerebral perfusion on ECCD (and TCCD), but EEG consistent with ILBF were found to have been hypoxic for > 7 days. ILBF had occurred > 1 week ago with subsequent cerebral reperfusion. The second patient was on ECMO therapy after CPR. This scenario is most likely to happen in patients with hypoxia and ILBF. Only around 10% of post-CPR patients develop cerebral edema with such an extensive mass effect that it causes subsequent herniation and whole brain death (29). In this small proportion of patients, cerebral reperfusion can occur after a reduction in cerebral edema. In this case, the sonographic signs of ILBF might be absent and other ancillary tests should be applied.

Regarding the flow patterns that are indicative of CCA, detailed criteria have been described for TC(C)D (21), but not for ECCD. However, if there is no net forward flow, the same flow signals that are compatible with CCA in intracranial vessel segments should also pertain to extracranial vessel segments. With regard to biphasic flow signals, it is important to heed (i) that the integral (area) of the ante- and retrograde segments of the Doppler frequency spectrum are equally sized and (ii) that only narrow, monophasic flow signals (orthograde component) should be accepted (21).

Some guidelines (including the GMA guideline) ask for the demonstration of CCA when the clinical examination (in particular the apnea test) cannot be completed or is inconclusive, i.e., in hypothermic patients, patients with relevant COPD or relevant levels of analgosedatives (30). This further corroborates the importance of simplifying cerebral vessel sonography for determining ILBF, particularly since alternative ancillary perfusion tests are more complex (CTA, DSA, SPECT) and require transport of critically ill patients to diagnostic facilities. In addition, the guideline-compliant protocol for these procedures is complex, fraught with pitfalls and not approved in some patient cohorts (e.g., CTA in patients < 18 years) (6, 31, 32).

In our experience, technical challenges pose a considerable hurdle in ancillary testing for ILBF and this pertains to both TCCD and EEG. In this study, TCCD was not helpful in one third of cases.

ECCD, however, can also be carried out with standard ultrasound machines and in the detection of the four extracranial brain-supplying arteries by ECCD is markedly easier in a technical sense than the detection of the intracranial arteries by TCCD in this patient cohort. Furthermore, and particularly if not done regularly, the recording of an EEG in the context of ILBF confirmation that meets the above-mentioned prerequisites can be difficult and precludes its application in many hospitals in our experience. ECCD however is a standard test that all neurologists and even many non-neurologists apply on a daily basis and the criteria that are required to confirm CCA do not pose any technical challenge. As a result, the diagnostic process for brain death is markedly simplified which facilitates end-of-life care and may help to identify more potential organ donors.

One possible limitation of our study might be that local regulations do not allow parts or all of the aforementioned ancillary tests in ILBF candidates, which limits the generalisability of the conclusions. This particularly pertains to the application of EEG, where its routine use as an ancillary test has been discouraged by the authors of the World Brain Death Project (4). Furthermore, in some patients, cerebral reperfusion, ECMO-therapy or osseus defects render ancillary tests for CCA inconclusive, which also pertains to the application of ECCD. Moreover, the sonographer in the present study was blinded to the result of the EEG, but not to that of the clinical examination. Our data needs to be confirmed in a larger, multicenter trial.

## Conclusions

ECCD performed more than 24 h after the first clinical signs of brain death yielded high levels of specificity and a high positive predictive value when compared to needle-electrode EEG, and could reliably demonstrate CCA in patients with ILBF. This potentially makes ECCD an alternative to currently established ancillary tests in this setting, but confirmation in a multi-center trial is warranted.

## Data availability statement

The original contributions presented in the study are included in the article/supplementary material, further inquiries can be directed to the corresponding author.

## Ethics statement

The studies involving human participants were reviewed and approved by Ethik-Kommission der Albert-Ludwigs-Universität Freiburg. Written informed consent to participate in this study was provided by the participants' legal guardian/next of kin.

## Author contributions

JL, CS, W-DN, and JB carry out the diagnostic tests in ILBF candidates on all ICUs at the University Hospital of Freiburg. JL, JB, and W-DN made substantial contributions to the study conception and design, as well as the acquisition, and analysis and interpretation of the data. JL has drafted the manuscript. CS, W-DN, and JB have revised it. All authors have read and approved the submitted final version of the manuscript.

## Conflict of interest

The authors declare that the research was conducted in the absence of any commercial or financial relationships that could be construed as a potential conflict of interest.

## Publisher's note

All claims expressed in this article are solely those of the authors and do not necessarily represent those of their affiliated organizations, or those of the publisher, the editors and the reviewers. Any product that may be evaluated in this article, or claim that may be made by its manufacturer, is not guaranteed or endorsed by the publisher.

## Abbreviations

AEP: acoustic evoked potentials
BA: basilar artery
CCA: cerebral circulatory arrest
CCT: cranial computed tomography
COPD: chronic obstructive pulmonary disease
CPR: cardiopulmonary resuscitation
CTA: computed tomography angiography
DGKN: Deutsche Gesellschaft für Klinische Neurophysiologie
DSA: digital subtraction angiography
ECCD: extracranial color-coded duplex sonography
EEG: electroencephalogram
GMA: German medical association
ICA: internal carotid artery
ICP: intracranial pressure
ICU: intensive care unit
ILBF: irreversible loss of brain function
MCA: middle cerebral artery
NPV: negative predictive value
PPV: positive predictive value
SPECT: single-photon computed tomography
SSEP: somatosensory evoked potentials
TCD: transcranial doppler sonography
TCCD: transcranial color-coded duplex sonography
VA: vertebral artery
vaECMO: veno-arterial extracorporeal membrane oxygenation.
